# Factors influencing quality of life, function, reintegration and participation after musculoskeletal tumour operations

**DOI:** 10.1186/s12885-020-06837-x

**Published:** 2020-04-25

**Authors:** Wolfram Weschenfelder, Sabine Gast-Froehlich, Christian Spiegel, Matthias Vogt, Gunther O. Hofmann

**Affiliations:** Department of Trauma, Hand and Reconstructive Surgery, University Hospital Jena, Am Klinikum 1, 07747 Jena, Germany

**Keywords:** Sarcoma, Quality of life, Depression, Anxiety, Operation, Amputation, Ablative surgery

## Abstract

**Background:**

The number of people living with soft-tissue and bone sarcomas is increasing due to improved individual therapy and changes in demographics. At present, there are no recommendations for psychological co-treatment, occupational and social reintegration following the treatment of soft tissue and bone sarcomas.

**Methods:**

Seventy-four patients, 42 males and 32 females, aged between 18 and 80 years (54.58 ± 16.99 yr.) with soft-tissue (62) and bone sarcomas (12) were included to answer five standardized and one personal questionnaire regarding quality of life, function, reintegration and participation after surgical treatment.

**Results:**

A number of tumour-specific and patient-specific factors were identified that affected the therapeutic outcome. Patients with sarcoma of the lower extremity described poorer mobility. Patients who underwent amputation reported a higher anxious preoccupation. Patients with a higher range of education were less fatalistic and avoiding. The size of tumours or additive radiation therapy did not affect the post-therapeutic quality of life, coping and function. There was a good correlation between anxiety and depression with occupational reintegration, function, quality of life and coping.

**Conclusion:**

Patients with sarcomas of the lower limb have a higher demand for postoperative rehabilitation and need more help in the postoperative occupational reintegration. Furthermore patients that underwent limb-preserving operations reported better postoperative function and quality of life. Risk assessment using patient-specific factors and an intensive psychological co-treatment may have a large role in the co-treatment of patients from the beginning of their cancer therapy.

## Background

Malignant diseases are the second most common cause of death in modern industrialized countries. The number of people living with cancer is increasing due to the demographic change and the extended survival of malignant tumours through continuous improvement of their therapy [1–3]. For instance, the five-year survival rate of patients with Ewing’s sarcoma was reported to be below 10% at the end of the 1970s and has recently been reported to be 50–80% in large studies [4, 5].

In 2013 cancer was the cause of an incapacity to work in around 200,000 cases in Germany and 1 in 8 nursing cases had a cancer disease as primary diagnosis [2]. Primary malignant bone and soft tissue tumours - the so-called bone and soft tissue sarcomas are rare types of tumours and account for only about 1% of cancers in adults. In children and adolescents, they account for approximately 15% of malignant tumours.

The treatment of sarcomas usually includes resection of the tumour-bearing tissue with a safety margin. This may have an effect on the function of a limb, the patient’s body image and thus the psychosocial outcome of the patient.

In addition to resection, factors such as the size of the tumour, the affected region or tissue type may also influence the postoperative outcome. Radiotherapy, isolated limb perfusion or chemotherapy may also be used as part of the therapy [6, 7]; their influence on the quality of life of sarcoma patients has not been clearly clarified. In addition, local surgical reconstruction may be performed very differently according to the surgeon’s preference. Bone defects must be reconstructed with autologous bone, foreign bone or prostheses. Muscle defects must be reconstructed with local or distant flaps in order to enable the best possible postoperative function [8]. Although the outcomes of reconstruction have improved, sometimes, an amputation is necessary, which still achieves good postoperative function due to modern external prosthesis.

In all malignant musculoskeletal tumours, a follow-up for the early detection of recurrence is suggested. However, we lack evidence that this has an influence on overall outcome and there is no recommendation regarding aid in occupational and social reintegration following the treatment of those malignancies. This is especially important in cases where treatment may be prolonged such as in cases of Ewing’s or osteosarcoma which may last almost 1 year [6, 7].

While the main focus is on the functional aspects of tumour surgery and oncological follow-up, the psychological diagnosis and adjuvant treatment is usually very under-represented in treatment guidelines. Occasionally, patients are hardly screened for existing disorders or symptoms are not properly recognized. Pirl et al. reported that 10 to 25% of cancer patients have concurrent depression or depressive disorders [9]. Similar results were reported by Trautmann et al. for patients with sarcomas with a prevalence of depression of 23–30% and anxiety disorders of 13–19% [10]. The influence of these psychological aspects on the overall outcome and the reintegration of patients after sarcoma operations have only been sparsely investigated so far.

Therefore, based on the above considerations, this study aimed to examine the following study questions:

1. Does the affected tissue type and the localization have an impact on subsequent occupational reintegration and quality of life?
2. Do the size of the tumour, use of radiation therapy and/or the surgical treatment have an influence on function, occupational reintegration and quality of life?
3. Does post-therapeutic function have an impact on quality of life and occupational reintegration?
4. Does the patient’s level of education have an impact on occupational reintegration, quality of life and mental adjustment to cancer?
5. Do patients with depressive or anxious traits experience poorer occupational reintegration, quality of life and adjustment to cancer?

## Methods

### Patients

This study is a retrospective analysis of 782 patients who underwent surgery for a musculoskeletal tumour including benign lesions and metastasis in the period from July 2004 to January 2018 at our institution.

Patients were included in this study if they were at least 18 years of age, were treated for a soft tissue or bone sarcoma, had the primary operative treatment in our institution and were at least 6 months after the completed cancer treatment including radiation therapy. Patients were excluded if they were treated in palliative intention, had a recurrence of the disease or a relevant secondary tumour.

Ninety-one of these patients were included in accordance with the inclusion and exclusion criteria. Seventy-four of these patients consented to participate in the study, completed the necessary questionnaires and could thus be included in the study.

### Materials

All patients were given a questionnaire, either by post or at the time of follow-up in the outpatient clinic, consisting of five standardized and one personal questionnaire; analysing school and vocational training, employment before and after the disease diagnosis, family support and residential situation. The standardized part of the survey included the following questionnaires in their validated German versions:

1. The SMFA (Short Musculoskeletal Function Assessment Questionnaire) is a practicable and reliable questionnaire for patient-centred assessment of musculoskeletal dysfunction consisting of 5 scales for function (Daily Activities, Emotional Status, Arm and Hand Function, Mobility) and 1 scale for bother [11, 12].
2. The HADS (Hospital Anxiety and Depression Scale) is a questionnaire for the assessment of anxiety and depression in patients with physical illnesses, developed by Zigmond and Snaith in 1983 [13, 14]. The state of the last week prior to questioning is recorded by seven items relating to anxiety symptoms and another seven items to record depressive symptoms arranged in alternating sequence. The generated scores can be inconspicuous, suspicious or conspicuous [15].
3. The QLQ-C30 (Quality of Life Questionnaire) is an instrument to measure quality of life developed by the European Organization for Research and Treatment of Cancer, applicable to all tumour entities and validated in numerous studies [16, 17]. A total score will not be earned - the scales and items must be considered individually [18].
4. The SF-36 (Short Form 36) is a tool for general health assessment based on the short form 20 scores of the Medical Outcomes Study in 1988 [19]. Using 36 different questions (items), the sheet determines statements about the health status of the patient over 8 different dimensions [20].
5. The last part of the questionnaire is the MAC (Mental Adjustment to Cancer), a self-assessment tool depicting various reactions to cancer like “Fighting Spirit”, “Anxious Preoccupation”, “Fatalism”, “Helpless−/Hopelessness” and “Positive Avoidance”. It is used specifically for the detection of disease processing in cancer patients (“coping”) [21]. Reliability and validity are sometimes rated as unsatisfactory, but sometimes as moderately to highly esteemed [22].

### Data analysis

Microsoft Excel 2013 (Microsoft Corporation, Redmond, Washington, USA) and IBM SPSS Version 25 (IBM Corporation, Armonk, New York, USA) were used for data analysis. For classified variables, the frequency and percentage of each pattern were calculated, and detected by a χ2 test according to Pearson. The measurement data showed no normal distribution so non-parametric tests had to be used to determine differences; the Mann-Whitney-U-test in case of two categories; the Kruskal-Wallis-test in case of more than two categories. The respective results were controlled by uni- and multivariate analysis (ANOVA and MANOVA). Metric variables were calculated using the Pearson Bivariate Correlation Test. In the text the respective median with inter quartile range is shown. A *p* < 0.05 was considered statistically significant.

## Results

The patient cohort consisted of 42 males and 32 females; aged between 18 and 80 years at the time of diagnosis, with average of 54.58 ± SD 16.99 yr. Sixty-two patients had a soft tissue sarcoma and twelve patients had a bone sarcoma. The distribution of tumour localization showed four tumours of the trunk, fifteen of the upper extremity and 55 of the lower extremity. Sixty-nine cases were limb preserving whilst five patients had to undergo an amputation of the diseased limb due to extent and localization of the tumour. Adjuvant Radiotherapy was used in 48 cases. 55.4% of the patients were in employment or training before diagnosis; 40.5% had already retired and the remaining 4.1% received an EU pension or were unemployed.

### Does the affected tissue type and the localization have an impact on subsequent occupational reintegration and quality of life?

**Fig. 1:**
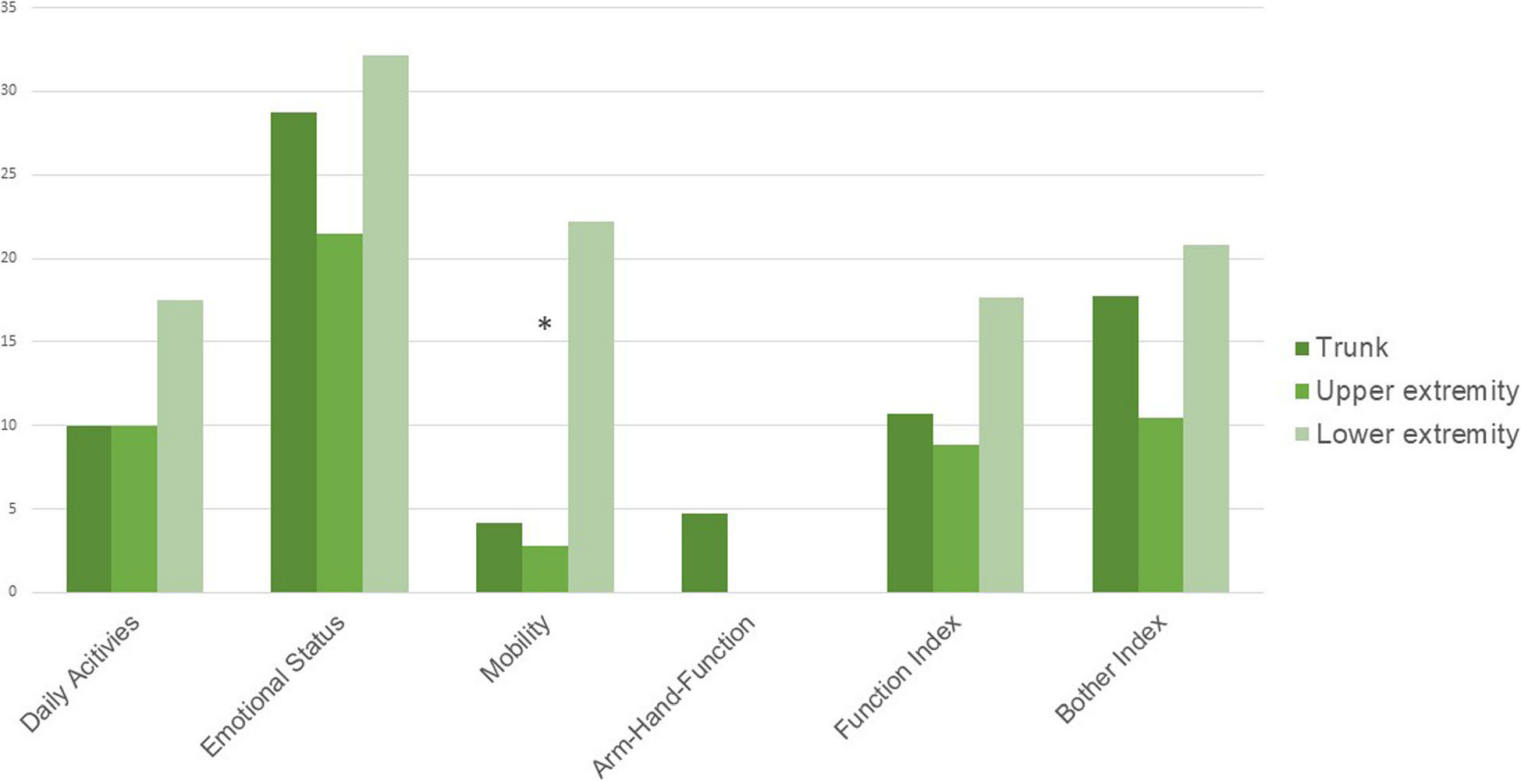
Median values of SMFA in relation to tumour localization

There was no correlation between tumour localization and occupational reintegration (*p* = 0.43). Analysis of tumour localization and musculoskeletal dysfunction measured by SMFA revealed a significant difference in the scale “mobility” (*p* = 0.015), having a median of 22.22 (5.56–44.44) in the “lower extremity” group, 4.17 (0.70–40.98) in “trunk” and 2.78 (0–13.89) in “upper extremity”. Similar results for the item “mobility” could be demonstrated in the multivariate analysis (MANOVA Wilks-Lambda *p* = 0.045, ANOVA mobility *p* = 0.022) (Fig. 1). There was no significant correlation of tumour localization on quality of life measured by SF-36 and QLQ-C30. Patients with tumours of the lower extremity had a significantly higher value of the item “Anxious Preoccupation” (*p* = 0.047) in the Kruskal-Wallis-test, being 21.00 (19.00–24.00) in median compared to 17.50 (16.25–22.50) for trunk and 19.00 (16.00–21.00) for upper extremity. These findings were however not significant in the multivariate analysis (MANOVA Wilks-Lambda *p* = 0.541, ANOVA anxious preoccupation *p* = 0.085).

In our patient cohort the affected tissue type (bone vs. soft tissue) had no significant influence on occupational reintegration, musculoskeletal dysfunction, quality of life or coping of tumour.

### Do the size of the tumour, use of radiation therapy and/or the surgical treatment have an influence on function, occupational reintegration and quality of life?

In the patient cohort no significant correlation between tumour size classified in groups according to the TNM-classification of soft-tissue sarcomas and outcome was found, in terms of occupational reintegration (*p* = 0.82), function by SMFA, quality of life using SF-36 and QLQ-C30 and coping with the disease by MAC. Furthermore, the influence of adjuvant radiotherapy on the occupational reintegration and the other measured parameters was not significant (*p* = 0.11).

When considering the mode of surgical reconstruction; whether limb preservation could be achieved or amputation was necessary, there was a significant impact on the occupational reintegration (*p* = 0.002). There were also significantly worse values in the amputation group for most of the functional and some related quality of life scores (SMFA: Daily Activities, Mobility, Mobility, Function Index, Bother Index; SF-36: Physical Functioning, Physical Role Functioning, Social Functioning; QLQ-C30: Physical Functioning, Role Functioning, Social Functioning, Financial Difficulties), as well as the “fatalism” item of the MAC that could be demonstrated using univariate and multivariate analysis (MANOVA Wilks-Lambda *p* = 0.003).

### Does post-therapeutic function have an impact on quality of life and occupational reintegration?

There was a direct linear relationship between the Functional Index of the SMFA and the perceived impairment, as measured by the Bother Index of the SMFA (*p* < 0.001).

Function as measured by the Function Index of the SMFA, also correlated with all relevant items of the SF-36. Analysis of the QLQ-C30 showed similar results, indicating a correlation between postoperative function and quality of life. There was a significant relation between coping with the disease and the Function Index, too (Table 1).

**Table 1:** p-Scores of correlation of Function Index with the items of SF-36, QLQ-C30 and MAC

SF-36	p	QLQ-C30	p	MAC	p
Physical functioning	< 0.001	Global Health	< 0.001	Fighting Spirit	0.025
Role functioning (physical)	< 0.001	Physical functioning	< 0.001	Anxious Preoccupation	0.006
Role functioning (emotional)	< 0.001	Role functioning	< 0.001	Fatalism	0.001
Energy / fatigue	< 0.001	Emotional functioning	< 0.001	Helpless−/Hopelessness	< 0.001
Emotional well-being	< 0.001	Cognitive functioning	< 0.001	*Positive Avoidance*	*0.85*
Social functioning	< 0.001	Social functioning	< 0.001		
Pain	< 0.001	Fatigue	< 0.001		
General Health	< 0.001	*Nausea and vomiting*	*0.27*		
*Health Change*	*0.081*	Pain	< 0.001		
		Dyspnoea	0.047		
		Insomnia	< 0.001		
		Appetite Loss	0.019		
		Constipation	0.027		
		*Diarrhoea*	*0.93*		
		Financial difficulties	< 0.001		

Finally, the relation between the different scores measuring function and occupational reintegration were analysed. The items “Daily Activity” (*p* = 0.001), “Emotional Status” (*p* = 0.001), “Mobility” (*p* = 0.002), Function Index (*p* = 0.001), Physical Functioning of the SF-36 (*p* = 0.003) and Physical Functioning of the QLQ-C30 (*p* = 0.003) showed a significant correlation with reintegration into professional life (MANOVA Wilks-Lambda *p* = 0.007).

### Does the patient’s level of education have an impact on occupational reintegration, quality of life and mental adjustment to cancer?

There was no significant relation between school education (*p* = 0.91) or vocational training (*p* = 0.12) and occupational reintegration.

**Fig. 2:**
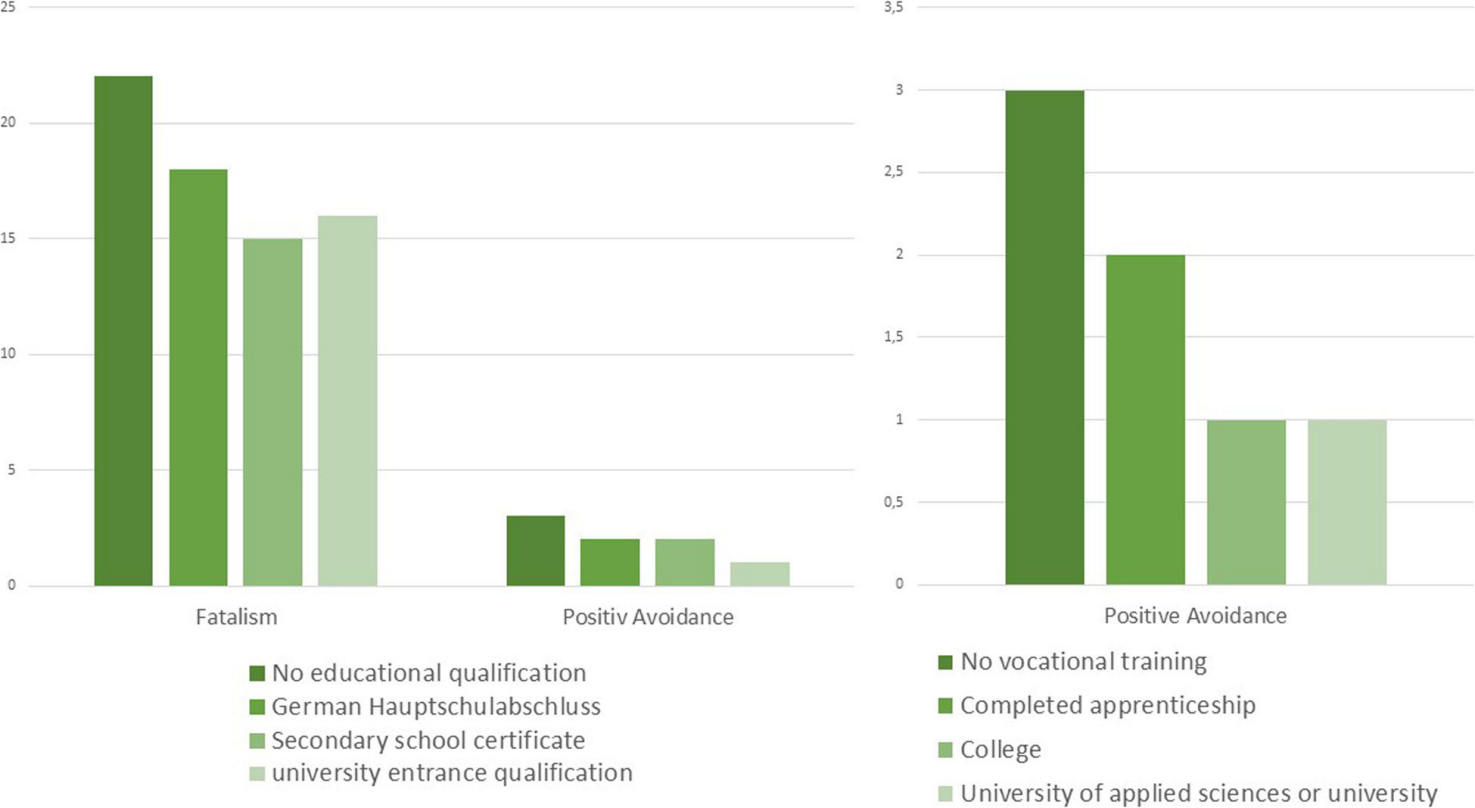
Median of significant items of MAC in relation to school education and vocational training

Looking at the influence on coping with the disease, measured by the MAC, significantly better results could be demonstrated for the items “Fatalism” (*p* = 0.026) and “Positive Avoidance” (*p* = 0.005) in patients with higher school education (MANOVA Wilks-Lambda *p* = 0.004). Similar results were found for vocational training for the item “Positive Avoidance” (*p* = 0.008, univariate analysis *p* = 0.017) (Fig. 2). No significant influence on quality of life (SF-36, QLQ-C30) was found either for school education or for vocational training.

### Do patients with depressive or anxious traits experience poorer occupational reintegration, quality of life and adjustment to cancer?

**Fig. 3:**
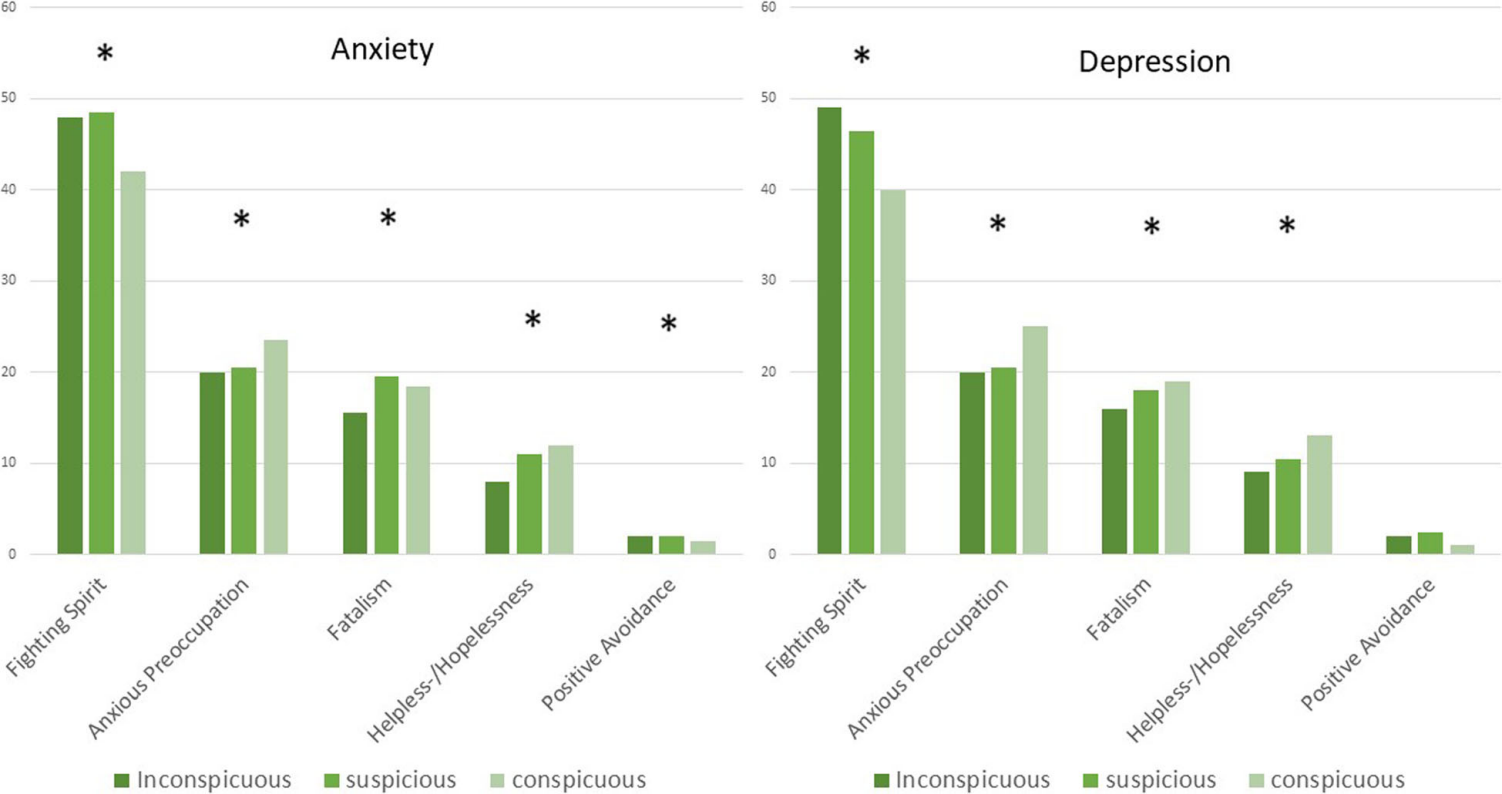
Median of MAC in correlation to groups of HADS regarding anxiety and depression

In the patient cohort there was a clear correlation between anxiety and coping with cancer (Fighting spirit *p* = 0.047, Anxious Preoccupation *p* = 0.005, Fatalism *p* = 0.010, Helpless−/Hopelessness *p* = 0.002, Positive Avoidance *p* = 0.047) as well as between depression and coping with the disease (Fighting spirit *p* < 0.001, Anxious Preoccupation *p* = 0.026, Fatalism *p* = 0.031, Helpless−/Hopelessness *p* < 0.001, Positive Avoidance *p* = 0.060). The relationships are presented in Fig. 3.

Occupational reintegration showed a significant correlation with the results of the HADS with respect to anxiety (*p* < 0.001) and depression (*p* = 0.002).

**Fig. 4:**
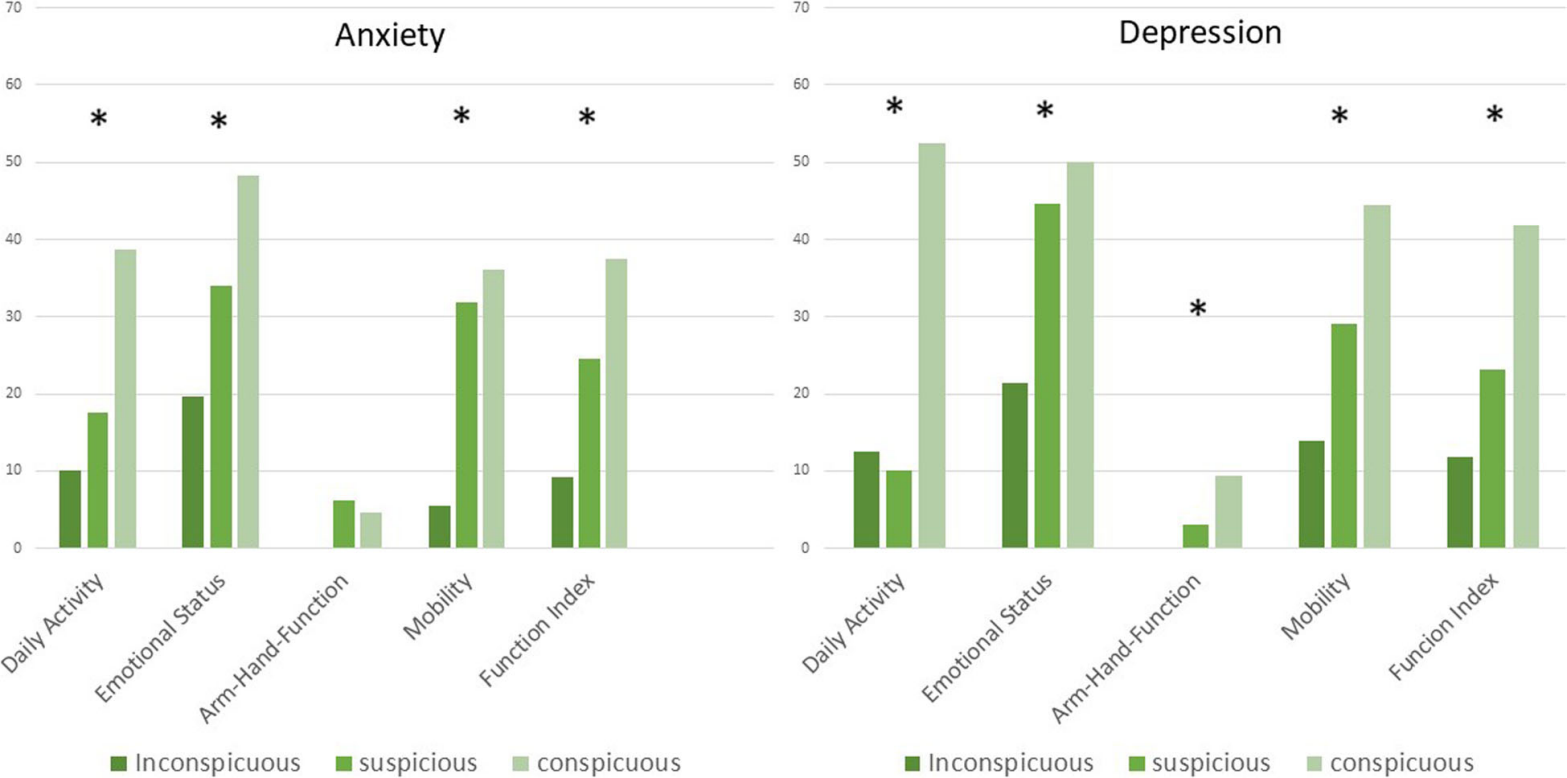
Median of function items of SMFA in correlation to HADS groups regarding anxiety and depression

There was a significant correlation between function assessed by SMFA and anxiety (Daily Activity *p* = 0.009, Emotional Status *p* < 0.001, Arm-Hand-Function *p* = 0.073, Mobility *p* < 0.001, Function Index *p* < 0.001) as well as depression (Daily Activity *p* < 0.001, Emotional Status *p* < 0.001, Arm-Hand-Function *p* = 0.014, Mobility *p* = 0.012, Function Index *p* < 0.001) measured by HADS (Fig. 4).

Considering the correlation between quality of life and anxiety as well as depression, there was a significant correlation for most items of the SF-36, Table 2.

**Table 2:** p-Scores of correlation of HADS-groups with the items of SF-36 and QLQ-C30

SF-36	p		QLQ-C30	p	
	Anxiety	Depression		Anxiety	Depression
Physical functioning	0.010	0.001	Global Health	< 0.001	< 0.001
Role functioning (physical)	0.003	< 0.001	Physical functioning	0.006	< 0.001
Role functioning (emotional)	< 0.001	< 0.001	Role functioning	0.007	< 0.001
Energy / fatigue	< 0.001	< 0.001	Emotional functioning	< 0.001	< 0.001
Emotional well-being	< 0.001	< 0.001	Cognitive functioning	< 0.001	< 0.001
Social functioning	< 0.001	< 0.001	Social functioning	< 0.001	< 0.001
Pain	< 0.001	0.004	Fatigue	< 0.001	< 0.001
General Health	< 0.001	< 0.001	*Nausea and vomiting*	0.13	0.25
Health Change	0.002	0.025	Pain	< 0.001	0.001
			Dyspnoea	0.002	0.047
			Insomnia	< 0.001	0.018
			*Appetite Loss*	0.065	0.13
			Constipation	0.005	0.49
			Diarrhoea	0.024	0.40
			Financial difficulties	< 0.001	< 0.001

## Discussion

### Clinical implications

The various factors influencing post-therapeutic outcome of patients with sarcomas can be differentiated in tumour-specific and patient-specific.

As expected, patients with sarcomas affecting the lower extremity described a poorer mobility than patients with tumours in other localizations and a higher anxious preoccupation. It appears that the size of the tumour and an additive radiation therapy do not affect the post-therapeutic quality of life, coping and function. The crucial factor is, whether a limb-preserving resection is possible or if an amputation is necessary. Patients following ablative surgery had a significantly worse function, quality of life and coping with cancer. There is a direct correlation between post-therapeutic function and post-operative quality of life as well as coping with the disease. These results underline the need for specific reconstructive surgery and intensive post-treatment.

Of the patient-specific factors, the patient’s educational background had an influence on coping with cancer. Patients with a higher level of education were less fatalistic and avoiding. Regarding anxiety and depression there was a clear correlation with all factors of the post-therapeutic outcome including occupational reintegration, function, quality of life and coping with the disease. Since the questionnaires examining these two traits were completed after the treatment, some of the results could be biased. However these findings highlight the importance of an intensive psychological co-treatment from the beginning of the cancer therapy.

### Study limitations

Limitations of the study are the relatively small number of patients and the resulting heterogeneity of individual groups. For example, only a distinction was made as to whether limb preservation or amputation took place - but not more precisely the surgical and reconstruction technique or the amputation level. This is due to the extreme rarity of sarcomas.

Another limitation is the studies retrospective design, which makes it difficult to evaluate the results of the survey on anxiety and depression. Finally, these could also be influenced by the existing poor function and restrictions in quality of life. Furthermore the questionnaires were completed at variable time points after treatment which might have an influence on the reported results.

## Conclusions

Patients with sarcomas of the lower limb have a higher demand for postoperative rehabilitation and need more help in postoperative occupational reintegration. Furthermore this study demonstrated that limb-preserving operations and a better postoperative function improve the quality of life of sarcoma patients. The results regarding patient-specific factors demonstrate that a survey of patient-specific risk factors including the social history and the expected coping options has to be carried out before the actual tumour therapy is initiated. An adapted intensive psychological co-treatment should be offered and implemented during the entire treatment process.

## Data Availability

The datasets used and/or analysed during the current study are available from the corresponding author on reasonable request.

## Abbreviations

SMFA: Short Musculoskeletal Function Assessment Questionnaire
HADS: Hospital Anxiety and Depression Scale
QLQ-C30: Quality of Life Questionnaire C-30
SF-36: Short Form 36
MAC: Mental Adjustment to Cancer
ANOVA: Analysis of the Variance
MANOVA: Multivariate Analysis of the Variance
